# Knockdown of PTOV1 and PIN1 exhibit common phenotypic anti-cancer effects in MDA-MB-231 cells

**DOI:** 10.1371/journal.pone.0211658

**Published:** 2019-05-13

**Authors:** Shibendra Kumar Lal Karna, Faiz Ahmad, Bilal Ahmad Lone, Yuba Raj Pokharel

**Affiliations:** Cancer Biology Laboratory, Faculty of Life Science & Biotechnology, South Asian University, Akbar Bhawan, Chankyapuri, New Delhi, India; University of South Alabama Mitchell Cancer Institute, UNITED STATES

## Abstract

**Background:**

Earlier, we have identified PTOV1 as a novel interactome of PIN1 in PC-3 cells. This study aims to explore the functional similarity and the common role of both genes in breast cancer cell proliferation.

**Methods:**

CTG, crystal violet assay, clonogenic assay, wound healing assay, cell cycle analysis, Hoechst staining and ROS measurement were performed to assess cell viability, colony forming potential, cell cycle arrest, nuclear condensation and ROS production after knocking down of PTOV1 and PIN1 by siRNAs in MDA-MB-231 and MCF-7 cells. CO-IP, qPCR and western blot were performedto study interaction, transcriptional and translational regulation of both genes.

**Results:**

Knockdown of PTOV1 and PIN1 inhibited the cell proliferation, colony formation, migration, cell cycle, and induced nuclear condensation as well as ROS production. Interaction of PTOV1 and PIN1 was validated by Co-IP in MDA-MB-231 cells. Genes involved in cell proliferation, migration, cell cycle, and apoptosis were regulated by PIN1 and PTOV1. PTOV1 knockdown inhibited Bcl-2, Bcl-xL and inducedBAX, LC3 and Beclin-1expression. Overexpression of PIN1 increased the expression of PTOV1. Knockdown of both genes inhibited the expression of cyclin D1, c-Myc, and β-catenin.

**Conclusions:**

PTOV1 and PIN1 interact and exert oncogenic role in MDA-MB-231 cells by sharing the similar expression profile at transcriptional and translational level which can be a promising hub for therapeutic target.

## Introduction

Breast cancer is the most common cancer occurring in women worldwide [1, 2]. Although there are many treatments available like hormone therapy, adjuvant therapy, and surgery, breast cancer remains a major challenge [3, 4]. Triple-negative breast cancer (ER, PR, and HER2/Neu negative) cases have poor prognosis and highlight the need to explore the new molecular targets for breast cancer therapy.

Protein-Protein interactions (PPIs) transduce many important cellular functions and their dysregulation can cause diseases. The expression of aberrant proteins seems to enhance their tumor-promoting function due to their interaction with their partners in the cancerous state [5]. Identification of cancer enabling PPI hubs that maintain or amplify the cell transformation potential in cancer is one of the major therapeutic strategies in the battle against cancer [6]. PIN1 is an established oncogene that regulates the fate of phosphorylated protein catalyzing cis-trans isomerization. PIN1 is overexpressed in breast cancer and mediates its function via RAS signaling, increasing the transcription of c-Jun towards Cyclin D1 [7]. Our previous study showed that PIN1 interacts with the novel protein Prostate Tumor Overexpressed 1(PTOV1) in PC-3 cells [8]. PTOV1 is a 46 kDa protein with a tandem duplication of two repeated homology blocks of the sequence of 151 and 147 amino acids closely related to each other, located on the 19q 13.3–13.4 chromosome. Chromosome 19 harbours a large number of genes modulated by androgens including PIN1. The overexpression of PTOV1 in prostate cancer may be due to the cumulative effect of genes residing on chromosome 19. The immunocytochemical analysis of PC-3 cell showed that PTOV1 is located in the cytoplasm close to the nucleus [9]. Overexpression of PTOV1 causes the expression of c-Jun both total and in phosphorylated form in prostate cancer cells. PTOV1 interacts with RACK1 to bind with 40s ribosomes during translation initiation stage [10].

The purpose of our study was to reveal how PTOV1 and PIN1 coordinate to drive breast cancer progression, towards this end we used siRNA silencing approach to find out the change in expression profile of various oncogenic signal molecules at transcription and translation levels in MDA-MB-231 cells. Targeting this complex can contribute to autophagy and apoptosis induced cell death increasing the efficacy of the therapeutic approach against breast cancer.

## Materials and methods

### Cell line, reagents, and antibodies

MDA-MB-231and MCF-7 breast cancer cell lines were purchased from NCCS, Pune, India. Lipofectamine RNAiMAX and Opti-MEM media were obtained from Invitrogen Corp (Carlsbad, CA, USA). siRNAs were purchased from Qiagen (Hilden, Germany). SYBR Green was obtained from Bio-Rad (Hercules, California). Cell culture media, trypsin, and antibiotics were purchased from HiMedia (France). Antibodies were purchased from Santa Cruz Biotechnology (Dallas, Texas, USA), and Cloud-Clone Corp. (Houston, USA). Cell Titer-Glo reagent was obtained from Promega Corp (Madison, Wisconsin, USA).

### Cell culture

MDA-MB-231and MCF-7 cells were cultured in the L-15 medium and Dulbecco’s Modified Eagle’s medium (DMEM) respectively containing 10% FBS (Fetal bovine serum), Penicillin (100 unit/ml) and Streptomycin (100μg/ml). The cell culture was incubated at 37°C in humidified air containing 5% CO_2_.

### Transfection

2–3ᵡ10 ^5^ cells/well were seeded in 6 well plates one day before siRNA transfection. 25 nM of each siRNA was mixed with 100 μl Opti-MEM media. Similarly, the complex of Lipofectamine RNAi max (4 μl/each well) and Opti-MEM (100 μl) was mixed well and incubated for 5 minutes at room temperature. After that siRNA and Lipofectamine RNAiMAX complexes in Opti-MEM were mixed in 1:1 proportion and incubated for 25 minutes at room temperature. Cells were transfected with siRNA complexes and incubated at 37°C for 72 hours.

### Cell viability assay

Cell viability was accessed by Cell Titer Glo (CTG) assay according to the manufacturer’s protocol. Briefly, MDAMB-231 cells were seeded in 96 well white culture plates at a density of 1000 cells/well in 180 μl of the medium. 20 μl of siRNA complexes of SCR(Scramble), PIN1, and PTOV1 was prepared as mentioned above and reversely transfected in cells. Cells were incubated at 37°C, 5% CO_2_ for 96 hours. Following incubation, 100 μl of media was added to each well and treated with 100 μl of CTG reagent and kept on a shaker for 2 minutes. The plates were kept at room temperature for 10 minutes to stabilize the luminescence signal. Luminescence was measured using microtiter plate ELISA reader (Bio Tek, Winooski, Vermont, USA).

We also, estimated the cell viability of MCF-7 by Crystal Violet assay after transfecting cells with scramble, PIN1 and PTOV1 siRNA. Briefly, 3000 cells/well were seeded in 96 well plates and on the next day cells were transfected with the siRNAs for 72 hours. Following incubation, cells were stained with 0.4% crystal violet in 50% methanol for 30 mins. After which wells were washed with tap water to remove the excess dye. 100 μl of methanol was put in each well to dissolve the dye attached to cells on a rocker for 20 mins. Then the absorbance was recorded using microtiter plate ELISA reader.

### Colony formation assay

MDA-MB-231 and MCF-7 cells were transfected with siRNAs for 48 hours. Then, they were collected and 1000 cells/well were seeded in 6 well plates. Cells were allowed to grow for two weeks till the colonies could be observed. The colonies were fixed with 3.7% formaldehyde for 30 minutes and stained with 0.4% crystal violet. Cells were washed with DPBS to remove excess dye. The plate was left to air dry and the colonies were counted using Image J software.

### Wound healing assay

3ᵡ10^5^ cells/well were seeded in 6 well plates and transfected with siRNAs. A scratch was made with 10 μl tip on the monolayer of cells at 72 hours post-transfection. Then they were washed with DPBS to remove detached cells. The images were captured using an inverted microscope at 0, 6, and 12 hours. The area of the wound was calculated using Image J software.

### Cell cycle analysis

2.5ᵡ10^5^ cells/well were seeded in 6 well plates one day before transfection. After which they were transfected with siRNAs and incubated for 48 hours. Following the incubation, cells were harvested and fixed with ice-cold 70% ethanol and kept overnight at 4°C. Next, they were washed with DPBS, collected, and centrifuged at 1500 rpm for 5 minutes, after which they treated with RNase A (50 U/ml) for 1 hour and stained with Propidium iodide (PI) (20 μg/ml). Cells were further subjected to cell cycle analysis by flow cytometry (BD FACS Verse.

### Hoechst staining

1.5ᵡ10^5^ cells were seeded in 6 well plates. Next day, cells were transfected with siRNAs and incubated for 72 hours. After which they were stained with Hoechst 33342 stain (2μg) and kept in the dark for 15 minutes. Next, they were washed with DPBS and the images were captured using Nikon fluorescent microscope.

### Measurement of the intracellular ROS production

Intracellular ROS generation was detected using fluoroprobe CM-H2DCFDA (Invitrogen). 1.5ᵡ10^5^ cells were seeded in 6 well plates and transfected with siRNAs for 72 hours. Cells were treated with 10 μM CM-H2DCFDA and kept in the dark at 37°C for 30 minutes. Then, they were stained with Hoechst 33342 and incubated for 15 minutes in the dark at room temperature. The wells were washed with DPBS and the images were captured using a fluorescence microscope.

### RNA extraction, cDNA preparation, and real time PCR

2ᵡ10^5^ cells were seeded in 6 well plates, transfected with siRNAs and incubated for 72 hours. Then cells were harvested for RNA isolation using TRIzol lysis reagent following the manufacturer’s protocol. 2μg of RNA was used for cDNA synthesis using RevertAid First strand cDNA synthesis kit (Thermo Fisher Scientific). Real time PCR was carried out using iTaq SYBR green mix (Biorad) in ABI 700, (Invitrogen). PCR cycle condition was as shown below.

Hold stage 95°c (10min), PCR Stage (40 cycles) 95°c (15 sec),60°c (1min) and melt curve stage 95°c (15 sec),60°c (1min),95°c (15 sec). Sequences of primers for Real Time PCR are; *PIN1* forward primer sequence 5’-TTTGAAGACGCCTCGTTTGC-3’, reverse primer sequence 5’-GTGCGGAGGATGATGTGGAT-3’, *PTOV1* forward primer sequence 5’-AGACACTGAAGAGCCTGTGC-3’, reverse primer sequence 5’-CGTTGACAAAGTTGCCCTGG-3’, *p21* forward primer sequence 5’CTGCCCAAGCTCTACCTTCC-3’, reverse primer sequence 5’-CGAGGCACAAGGGTACAAGA-3’, *p27* forward primer sequence 5’-TGCAACCGACGATTCTTCTACTCAA-3’, reverse primer sequence 5’-CAAGCAGTGATGTATCTGATAAACAAGGA-3’, *SURVIVIN* forward primer sequence 5’-GGACCACCGCATCTCTACAT-3’, reverse primer sequence 5’-GAAACACTGGGCCAAGTCTG-3’, *NANOG* forward primer sequence-5’-GGACCACCGCATCTCTACAT-3’, reverse primer sequence-5’-TGCAGAAGTGGGTTGTTTGC-3’, *CYCLINB1* forward primer sequence-5’-AGGCGAAGATCAACATGGCA-3’, reverse primer sequence-5’-AGGCGAAGATCAACATGGCA-3’, *CYCLINE1* forward primer sequence-5’- ATACTTGCTGCTTCGGCCTT-3’, reverse primer sequence-5’- TCAGTTTTGAGCTCCCCGTC-3’, *CYCLINA1* forward primer sequence-5’-GAAAATGCCTTCCCTCCAGC-3’, reverse primer sequence-5’-TGTGCCGGTGTCTACTTCAT-3’, *CDK1* forward primer sequence-5’-TACAGGTCAAGTGGTAGCCA-3’, reverse primer sequence-5’-AGCACATCCTGAAGACTGACT-3’, *mTOR* forward primer sequence-5’-GCAGAAGGTGGAGGTGTTTG-3’, reverse primer sequence-5’-CATTGACATGACCGCTAAAGAACG-3’, *SOX2* forward primer sequence-5’-TTTGTCGGAGACGGAGAAGC-3’, reverse primer sequence-5’-TAACTGTCCATGCGCTGGTT-3’, *SLUG* forward primer sequence-5’-ACGCCTCCAAAAAGCCAAAC-3’, reverse primer sequence-5’-ACTCACTCGCCCCAAAGATG-3’, *c-Myc* forward primer sequence-5’-CCCTCCACTCGGAAGGACTA-3’, reverse primer sequence-5’-GCTGGTGCATTTTCGGTTGT-3’, *RACK1* forward primer sequence-5’-ATGGGATCTCAACGAAGGCA-3’, reverse primer sequence-5’-CACACAGCCAGTAGCGGTTA-3’, *CIAP* forward primer sequence-5’- AAGGAGTCTTGCTCGTGCTG-3’, reverse primer sequence-5’AGCATCAGGCCACAACAGAA-3’, *E2F1* forward primer sequence-5’- ACTCAGCCTGGAGCAAGAAC-3’, reverse primer sequence-5’GGTGGGGAAAGGCTGATGAA-3’, *PCNA* forward primer sequence-5’- TCTGAGGGCTTCGACACCTA-3’, reverse primer sequence-5’- CATTGCCGGCGCATTTTAGT-3’, and *GAPDH* forward primer sequence-5’-GTGAACCATGAGAAGTATGACAAC-3’, reverse primer sequence-5’-CATGAGTCCTTCCACGATACC-3.

### Western blotting

2ᵡ10^5^ cells were cultured in 6 well plates. Next day, cells were transfected with siRNAs and incubated for 72 hours. Following incubation, cells were washed twice with ice-cold DPBS, and the cell lysate was prepared using sodium dodecyl sulphate (SDS) lysis buffer. The samples were heated for 5 minutes at 100°C in a dry bath, run on SDS-PAGE, and then transferred to PVDF membrane (100 volts, 1 hour). The membranes were incubated with 5% skim milk in Tris buffer saline with 0.1% Tween20 (TBST) for 2 hours at room temperature. The blots were further incubated with primary antibodies and kept overnight at 4°C on a rocker. Then the blots were washed 5 times with TBST and probed with horseradish peroxidase (HRP) conjugated secondary antibodies. The blots were exposed to X-ray film in the dark using enhanced chemiluminescence (ECL, Biorad). β actin was used as a loading control.

### Co-Immunoprecipitation

The MDA-MB-231 cells were washed with cold PBS 2 times and collected in PBS using a scrapper. Cells were centrifuged at 1000 rpm for 5 minutes. Cells were lysed in lysis buffer (50 mM Tris (pH 7.5), 5mM EDTA, 150 mM NaCl, 1 mM dithiothreitol, 0.01% Nonidet P-40, 0.2 mM phenylmethylsulfonyl fluoride, and 1X protease inhibitor mixture). Cells were sonicated for 10 seconds. Cells were again centrifuged for 15,000 rpm for 20 minutes at 4°C.The supernatant was collected in an Eppendorf tube. 50μl of lysate was collected for input. Remaining cell lysate was divided into two equal parts in 2 Eppendorf tube and made 1 ml using lysis buffer and labelled as PIN1 IP and normal rabbit IgG. The lysates were incubated with 2 μg of PIN1 and normal rabbit IgG in respective tubes at 4°C with gentle rolling for 12–24 hours. On the next day, homogenous solution of magnetic beads was taken in two Eppendorf tubes and placed in a magnet. The supernatant was thrown. The lysates containing antibody complex were incubated with the beads for 1hour at 4°C with gentle rolling. Again, the tubes were placed in the magnet and the supernatant was thrown. The beads were washed with 200μl of washing buffer for 5 times by gentle pipetting with ice incubation for 30 seconds in each wash. The tubes were placed in a magnet and the supernatant was thrown. This was repeated for 5 times. The samples along with input were heated with 2XSDS sample buffer for 5 minutes at 100°C. The samples were again placed in the magnet and the supernatant was collected in new tubes. The input (2% of lysate volume), PIN1 IP and normal rabbit IgG Samples were loaded on 10% SDS PAGE and subjected to electrophoresis [8]. Immunoblotting was done with PIN1 and PTOV1 antibody and developed using ECL.

### Statistical analysis

Results are represented as a mean ± standard deviation. The level of significance between scramble (control) groups and the tests (PIN1 and PTOV1 siRNA treated) groups have been calculated by *ONE WAY ANOVA* and students’s t-test. P < 0.05 was considered statistically significant. *, **, *** represents P< 0.05, 0.01 and 0.001 respectively.

## Results

### Silencing of PTOV1 and PIN1 suppress the cell viability of MDA-MB-231 cells

**Fig 1:**
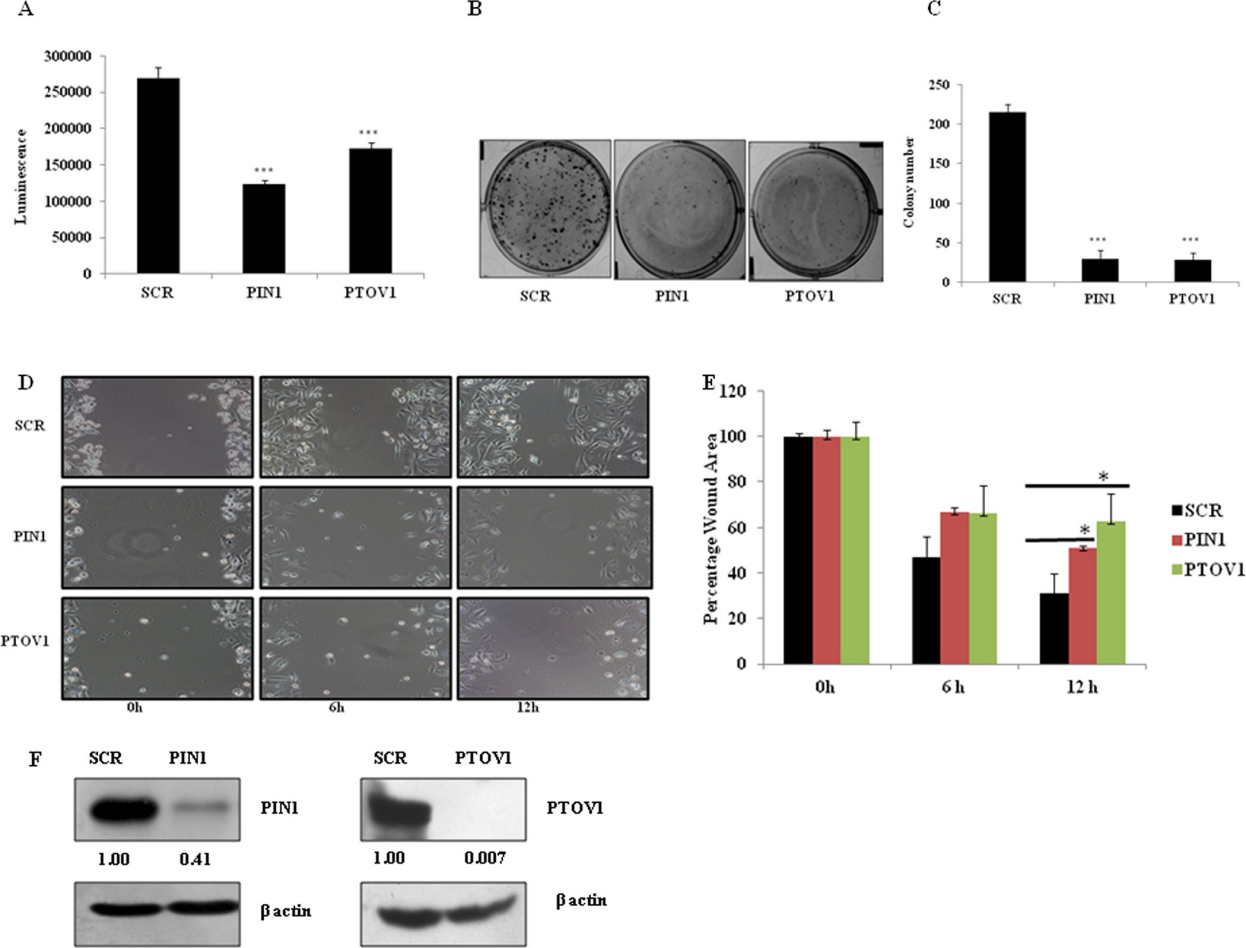
Silencing of PIN1 and PTOV1 decrease the proliferation, colony formation, and migration of MDA-MB-231 cells. (A) Cell viability was estimated by using CTG assay and luminescence signal was recorded in microtiter plate ELISA reader. (B) Representative images of the colony of cells transfected with siRNAs in 6 well plates after 2 weeks of incubation. (C) Densitometry representation of colony number using Image J software. (D) Representative images of the wound area of each treated group after 0, 6, and 12 hours. (E) Densitometry representation of Wound area using Image J software. (F) Representative image of PIN1 and PTOV1protein knockdown after transfecting cells with respective siRNAs. β- actin was used as a loading control. Data are represented as mean ± SD of three independent experiments. *** Significant difference from scramble groups (p < 0.001).

Cells were knockdown for PIN1 and PTOV1 genes by transfecting with siRNAs and incubated for 96 hours. Cell viability was estimated by using CTG reagent which measures the level of ATP present in the sample which indicates cell viability. The cell viability of MDA-MB-231 was significantly inhibited after silencing of PIN1 and PTOV1 genes by siRNAs in MDA-MB-231 cells (Fig 1A). The result suggested that both the genes are essential for the proliferation of MDA-MB-231 cells. Similarly, we have performed crystal violet assay to test cell viability in MCF-7 cells after transfecting with siRNAs. The results showed similar effect in MCF-7 cells as well (S1A Fig). As shown in Fig 1F, western blot images of PIN1 and PTOV1 knockdown by their respective siRNA are shown.

### Silencing of PTOV1 and PIN1 inhibit colony formation of MDA-MB-231 cells

To study the long-term effect of the silencing of PIN1 and PTOV1 on cell proliferative potential, we performed colonogenic assay which is associated with tumor formation *in vivo* [11]. Briefly, cells were transfected with siRNAs for 48 hours, collected, and 1000 cells per well were seeded in 6 well plates. Cells were allowed to grow for 14 days. The number and size of colonies were significantly inhibited in PIN1 and PTOV1 knockdown sample as compared to scramble (p< 0.001) (Fig 1B). Individual colonies were counted using Image J software and densitometry analysis was done (Fig 1C). Thus, silencing of both genes inhibited the proliferation and colony formation of MDA-MB-231 cells. As shown in S1B & S1C Fig, similar effect has been seen in MCF-7 cells with PIN1 and PTOV1 knock down by siRNA transfection. Colony numbers and size in PIN1 and PTOV1 knockdown cells were significantly inhibited as compared to scramble.

### Silencing of PTOV1 and PIN1 attenuate the migratory potential of MDA-MB-231 cells

The migratory behaviour of a cell is an important parameter in studying the invasive and metastatic potential following the treatment. Wound healing assay is a conventional technique to study the migratory behaviour of a cell [12]. In our experiment, we made a scratch on the monolayer of cells previously transfected with the above-mentioned siRNAs. The images were captured at 0, 6, and 12 hours using an inverted microscope. We observed that cell migration was significantly attenuated in PIN1 and PTOV1 knockdown samples as compared to scramble (Fig 1D). Quantitative analysis of the wound area using Image J software showed significant difference between scramble and PIN1 and PTOV1 knockdown cells at 12hr (p< 0.05) (Fig 1E). These results suggested that both the genes might be crucial for the migration of cells. In similar manner, we studied migration potential of MCF-7 cells and found that migration was significantly inhibited in 24 and 48 hours after knocking down of PIN1 and PTOV1 as shown in S1D & S1E Fig. Thus, the result showed both PIN1 and PTOV1 genes are important for migration of MCF-7 cells.

### Silencing of PTOV1and PIN1 induce G2/M phase arrest of MDA-MB-231 cells

**Fig 2:**
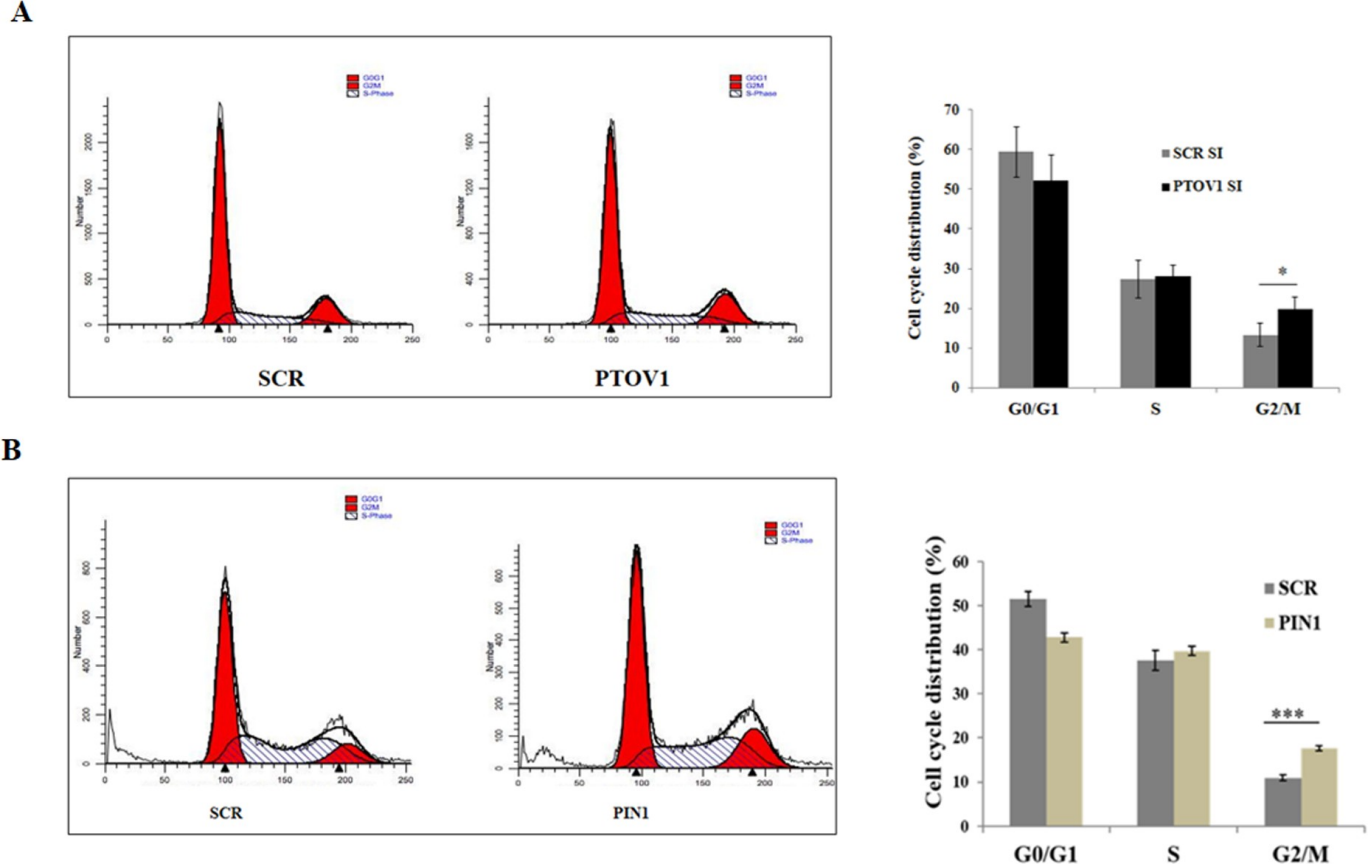
Silencing of PTOV1 and PIN1 lead to the G2/M arrest in MDA-MB-231 cells. (A) Histogram representing the cell cycle distribution of MDA-MB-231 cells transfected with PTOV1 siRNA for 48 hours (left panel) and statistical representation of percentage distribution of the population in each phase (right panel). (B) Histogram representing the cell cycle distribution of MDA-MB-231 cells transfected with PIN1siRNA for 48 hours (left panel) and statistical representation of percentage distribution of the population in each phase (right panel). Data are represented as mean ± SD of three independent experiments. *, *** Significant difference from scramble groups (p < 0.05 & 0.001).

After 48 hours of knocking down of cells with PIN1 and PTOV1 siRNAs, cells were subjected to cell cycle analysis by flow cytometry. Briefly, cells were collected, fixed with ice-cold 70% ethanol and incubated overnight at 4°C. This was followed by RNase treatment and staining with Propidium Iodide (PI). Percentage distribution of the cell in different phases of the cell cycle was estimated using flow cytometry which showed that there was a significant proportion of cells in G2/M phase for PTOV1 siRNA treated groups (19.73%) as compared to that in scramble siRNA treated groups (13.266%) (p < 0.05) (Fig 2A). The results showed that silencing of PTOV1 has an impact on cell cycle, but the effect was moderate. Knockdown of PTOV1 might block the entry of cells into the mitotic phase. We have also performed cell cycle analysis after 48 hours of knocking down of PIN1 in MDA-MB-231 cells. Result showed that PIN1 knockdown arrests cells in G2/M phase significantly showing similar effect as PTOV1[scramble (10.89%) & PIN1(17.65%)]. (Fig 2B)

### Silencing of PTOV1 and PIN1 induces nuclear condensation and initiation of apoptosis

**Fig 3:**
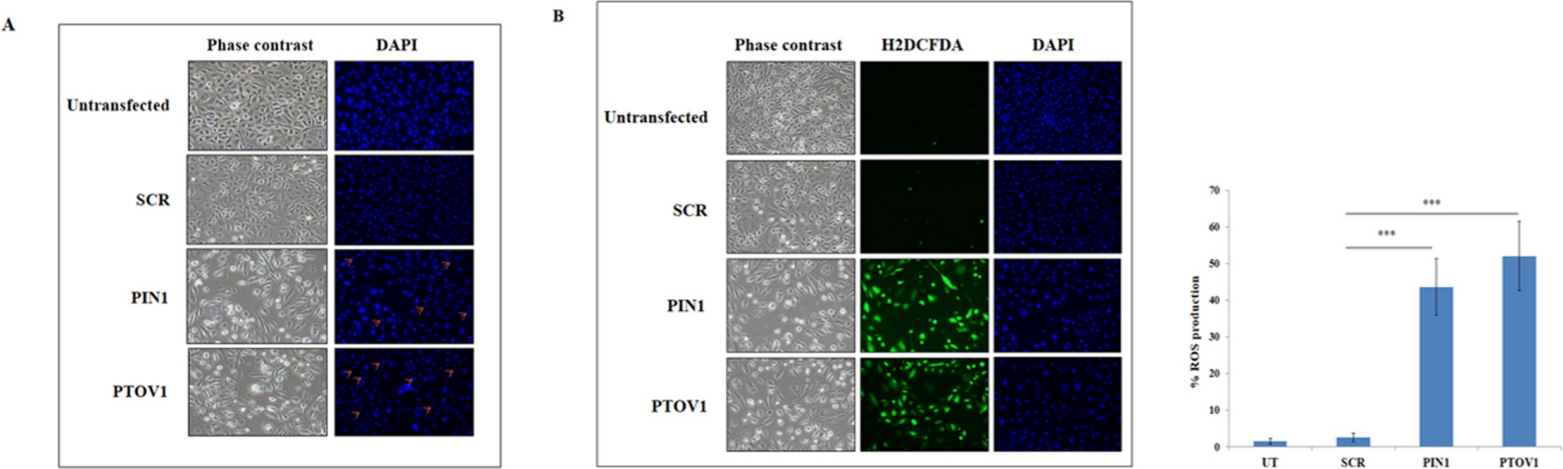
Silencing of PIN1 and PTOV1 alter cell morphology, apoptosis, and intracellular ROS production. (A) Representative images of cells transfected with the siRNAs after 72 hours and stained with Hoechst 33342. (B) Microscopic evaluation of intracellular ROS production in each group after 72 hours using CM-H2DCFDA. Images were captured using Nikon fluorescent microscope, right panel shows the graphical representation of the ROS production in different set of experiments. *** represents the significant difference from scramble (p< 0.001).

Nuclear condensation is a phenotype which indicates the initiation of apoptosis [13, 14]. Cells were transfected with the siRNAs for 72 hours and then stained with Hoechst 33342 dye. The phase contrast images showed that the morphology of cells in PIN1 and PTOV1 samples were altered and not healthy as compared to untransfected and scramble and the DAPI images showed that the nuclear size was reduced in knockdown samples compared to scramble and untransfected cells. These results showed that silencing of both the genes might initiate cells towards apoptosis (Fig 3A).

### Silencing of PTOV1and PIN1 elevate the intracellular ROS production

The level of ROS production has a determinant role on the behaviour of cells. In the context of cancer, it possesses both an oncogenic property sustaining the proliferation of cancer cells as well as a tumor suppressor property when there is an aberrant increase in ROS due to any kind of stimuli [15]. Thus, the search for the genes modulating the redox status of a cancer cell can be a good anti-cancer strategy [16]. As shown in Fig 3B, there was an increased production of ROS in PIN1 and PTOV1 silenced cells compared to scramble siRNA and non-transfected cells. Bar graph in right panel showed the quantification of ROS production by counting number of cells producing ROS out of total number of cells. Thus, our results revealed that downregulation of PIN1 and PTOV1 modulate the redox status of MDA-MB-231 cells enhancing their death significantly.

### Silencing of PTOV1 and PIN1 initiates the cell death of MDA-MB-231 cells by inducing apoptosis

**Fig 4:**
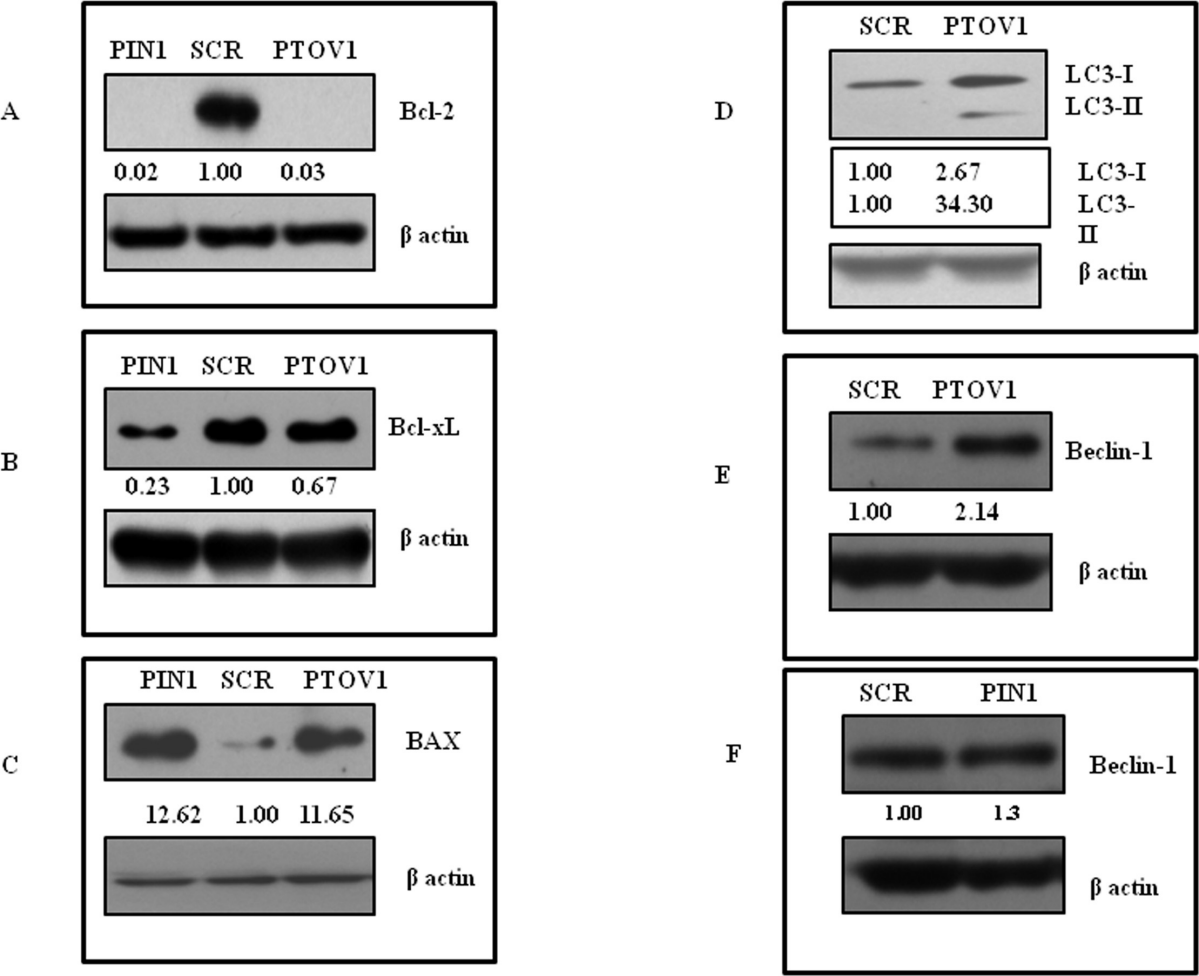
Silencing of PIN1 and PTOV1 drive cells towards apoptosis and PTOV1 knockdown further induces autophagy.Western blot analysis of (A) Bcl-2, (B) Bcl-xL, and (C) BAX after 72 hours of transfection. Expression analysis of autophagy markers (D) LC3, and (E) Beclin-1after transfecting cells with scramble and PTOV1 siRNA for 72 hours (F) Beclin-1 after transfecting cells with respective scramble and PIN1 siRNAs. The density was calculated by image J software. β- actin was used as a loading control. The experiments were performed in triplicates.

To study the cell death mechanism initiated by knockdown of PIN1 and PTOV1, western blot analysis was carried out to check the expression of apoptosis-related genes. There was inhibition of anti-apoptotic Bcl-2 (Fig 4A), Bcl-xL (Fig 4B), and up-regulation of pro-apoptotic BAX (Fig 4C). These results clearly suggested that inhibition of both the genes initiate cell death through apoptosis. Also, we found the enhanced expression of autophagy markers LC3 (Fig 4D) and Beclin-1(Fig 4E) after PTOV1 (Beclin-1 was unaffected by PIN1 knockdown (Fig 4F), These results demonstrated that PTOV1 silencing also induced cell death by autophagy.

### Silencing of PTOV1 and PIN1 share regulatory networks and affect diverse cellular functions at the transcriptional level

**Fig 5:**
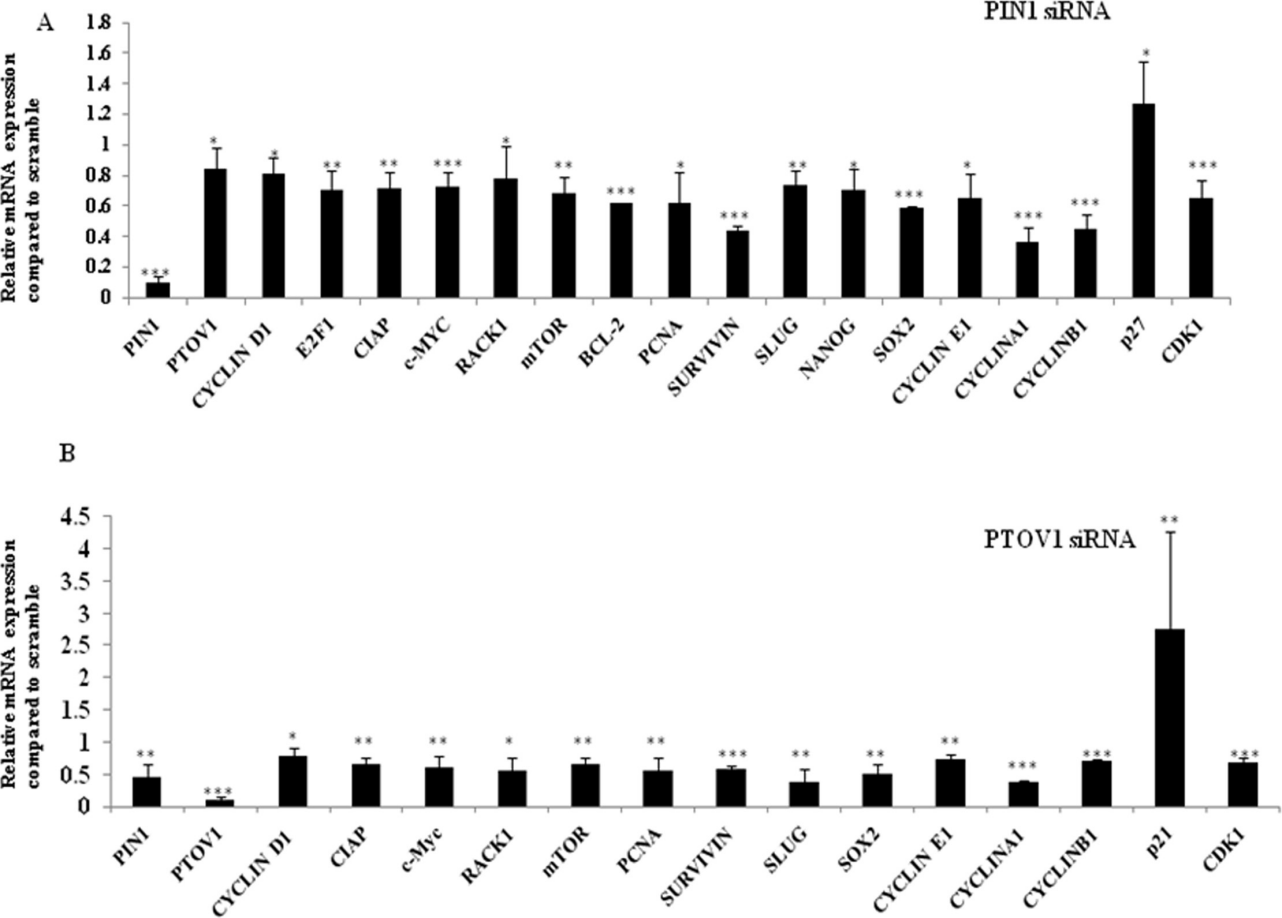
Silencing of PIN1 and PTOV1 share similar expression profile for the majority of genes at the transcriptional level. (A) The upper panel represents the expression of mRNA after transfecting cells with PIN1 siRNA for 72 hours. (B) The lower panel represents the expression of mRNA after knockdown with PTOV1 siRNA for 72 hours. Relative expression was calculated using delta-delta CT method. The expression in scramble group was considered 1.00. Experiments were performed in triplicates. Data are represented as mean ± SD.*, **, *** represents significant difference from the scramble with p< 0.05, 0.01 and 0.001 respectively.

mRNA expression of the genes affecting cell proliferation, cell cycle, apoptosis, and metastasis was studied after knockdown of cells with siRNAs for 72 hours. As shown in Fig 5A & 5B, the bar graph showed the relative expression of mRNAs in PIN1 and PTOV1 siRNA transfected samples compared to scramble samples respectively. The value for the expression of genes in scramble sample is considered to be 1.00.

Study of the regulation of various genes by PIN1 and PTOV1 at transcriptional level mentioned in Table 1, we realized that knockdown of both the genes shared similar expression profile and affect the major functions required for cancer cell progression. The expression was downregulated for cell proliferation, cell cycle, and metastasis-related genes while cell cycle inhibitor p27 and p21 were upregulated in PIN1 and PTOV1 siRNA transfected samples respectively. Although P53 is important for the transcription of p21 and its mutation is associated with poor p21 expression, the p53-independent expression of p21 has also been investigated. p21 interacts with the PCNA that drives the inhibition of DNA synthesis and arrests the cell cycle progression of p53 mutant cells in G1 and G2 [29–31]. Our findings are consistent that knockdown of PTOV1 downregulates the expression of PCNA at mRNA level. PIN1 is involved in releasing CDK2 by inhibiting P27 to arrest cell cycle in G1/S transition [32]. PIN1 regulates different cell cycle regulatory proteins including P27 which is one of the target of PIN1 [33]. Our finding suggests that one of the important cyclin dependent kinase inhibitor P27 induced with PIN1 knockdown.

**Table 1:** The genes used for the mRNA expression analysis with their cellular functions are listed in the table below.

Genes	Functions	References
1. PIN1	Cell proliferation, cell cycle	[7]
2. PTOV1	Cell proliferation	[9]
3. E2F1	Transcription factor, cell cycle	[17]
4. CIAP	Cell survival	[18]
5. c-Myc	Transcription factor, cell proliferation	[19]
6. RACK1	Adaptor protein, pro- or anti-oncogenic effect	[20, 10]
7. mTOR	Cell proliferation and survival	[21]
8. PCNA	DNA replication and repair	[22]
9. SURVIVIN	Cell proliferation and metastasis	[23]
10. SLUG	EMT and Cell survival	[24]
11. NANOG	Development and malignant progression of tumors	[25]
12. SOX2	Development and malignant progression of tumors	[25]
13. CYCLIN E1	Partner of cdk2, Important for the G1-S transition.	[26]
14. CYCLIN A1	Binds to E2F1 and Retinoblastoma (RB). High expression during S and G2/M phase.	[27]
15. CYCLIN B1	Binds with CDK1 and required for G2/M transition	[28]
16. p21	Inhibitor of cyclin E/cdk2 complexes,	[26]
17. p27	Inhibitor of cyclin E/cdk2 complexes,	[26]
18. CDK1	Partner with A and B type cyclins. Required for progression through mitotic phase	[26]

Our results suggest that a wide range of genes which are involved in cell proliferation, cell cycle, metastasis, and apoptosis are regulated by PIN1 and PTOV1 at the transcriptional level significantly.

### PTOV1 interacts with PIN1 and share the regulatory network of multiple oncogenic signaling pathways at the translational level

**Fig 6:**
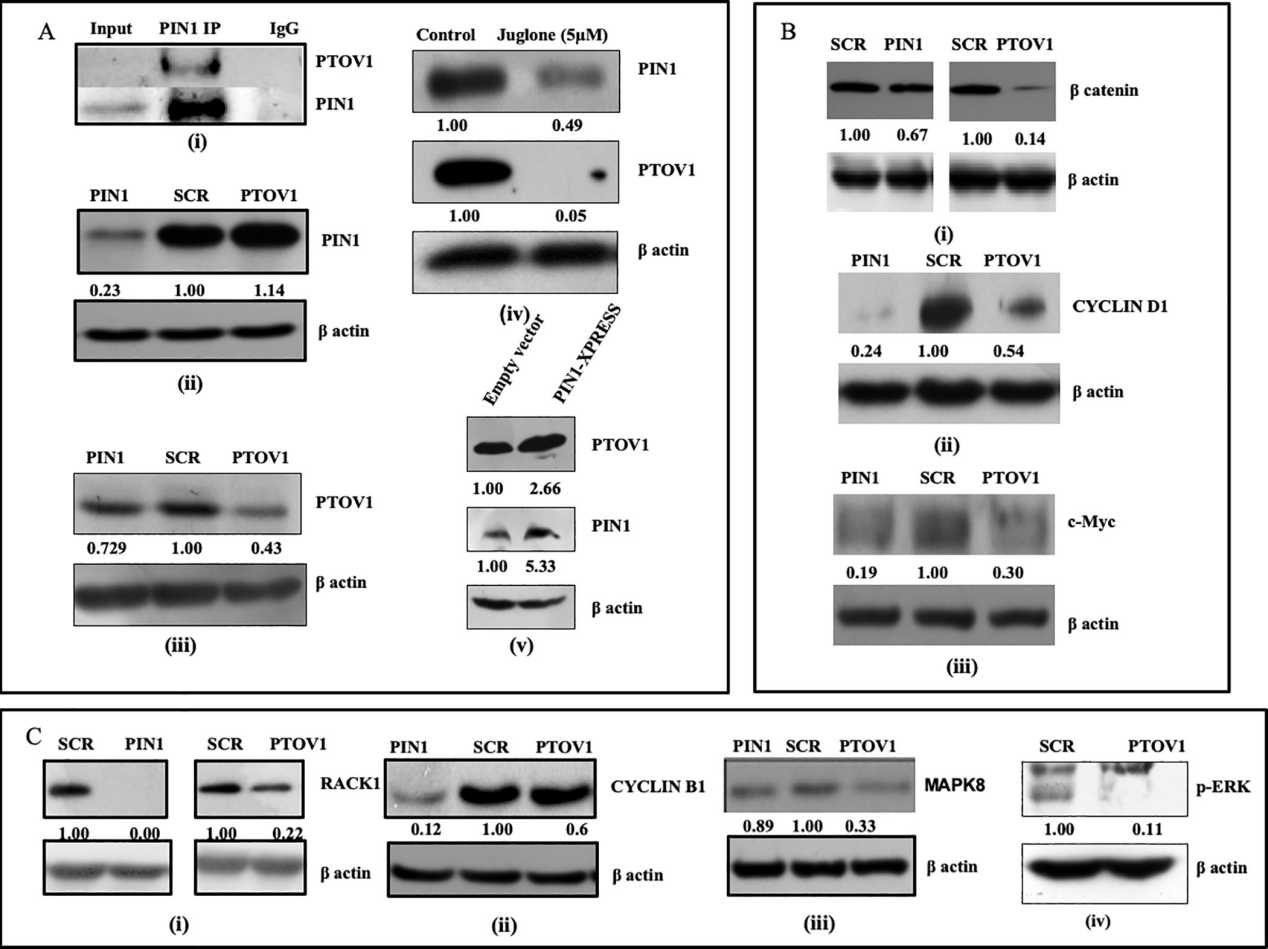
Silencing of PIN1 and PTOV1 cooperatively target multiple proteins and share similar expression status at the translational level. A. (i) PIN1 and PTOV1 interaction studied by co-immunoprecipitation in MDA-MB-231 cells. (ii& iii) Western blot analysis of PIN1 and PTOV1 after transfecting cells with siRNAs for 72 hours, (iv) with Juglone (5 μM) for 48 hours, and (v) Overexpression with PIN1-XPRESS vector. Expression analysis of B. (i) β- catenin, (ii) Cyclin D1, (iii) c-Myc, & further analysis of expression of C. (i) RACK1, (ii) Cyclin B1, (iii) MAPK8, and (iv) p-ERK after 72 hours of siRNA transfection. The densitometry was calculated by using image J software. β-actin was used as a loading control. The experiments were performed in triplicates.

We have already reported that PIN1 interacts with PTOV1 in PC-3 cells [8]. We have performed Co-IP with PIN1 IP to see the interaction of PIN1 and PTOV1 in MDA-MB-231 cells. As shown in Fig 6A. (i) PTOV1 is enrich in PIN1 complex. Furthermore, we analyzed the common regulatory network of both the genes at the protein level Western blots were performed after transfecting cells with siRNAs for 72 hours. Knockdown of PIN1 inhibited the expression of PTOV1 but there was no change in the expression of PIN1 protein level with PTOV1 knockdown Fig 6A (ii & iii). Cells were also treated with Juglone (5μM), a PIN1 inhibitor, for 48 hours. The expression of both PIN1 and PTOV1 was inhibited significantly Fig 6A (iv). These results suggested that PTOV1 contributes for PIN1 mediated MDA-MB-231 cell proliferation. Moreover, the overexpression of PIN1 with PIN1-XPRESS increased the expression of PTOV1 more than two folds Fig 6A (v). These results supported our previous finding that PTOV1 and PIN1 interaction play an important role in cancer cell proliferation [8].

PIN1 and PTOV1 silencing inhibited the expression of β-catenin Fig 6B (i) which is involved regulates the expression of both Cyclin D1 and c-Myc Fig 6B (ii, & iii). We have earlier shown that PTOV1 regulates the PIN1 substrate c-Jun phosphorylation [8]. These data supported our finding that PIN1 and PTOV1 contribute to oncogenesis of breast cancer cells targeting Cyclin D1 and c-Myc expression. PTOV1 interacts with the RACK1 to bind with the 40s ribosomes during translation initiation [10]. RACK1 has earlier been shown to be the regulator of cell signaling, migration and ribosomal functioning [34–36]. In our experiment, we found that PIN1 and PTOV1 knockdown suppressed the expression of RACK1. Thus, our results showed that PIN1 is required for PTOV1 mediated translational machinery through RACK1 Fig 6C (i). We also checked the expression of Cyclin B1, a G2/M transition regulatory protein and found that its expression was inhibited by siRNAs Fig 6C (ii). This result supported the role of PTOV1 in cell cycle regulation. The expression of MAPK8 (c-Jun N-terminal kinase) which phosphorylates c-Jun Fig 6C (iii) was also inhibited. ERK is already known to be the substrate of PIN1 [37]. Decreased phosphorylated ERK by PTOV1 downregulation suggested that PIN1 and PTOV1 share the similar expression profile of MAPK pathway Fig 6C (iv). These results showed the mechanisms of the PIN1 and PTOV1 cascade involvement in breast cancer cell proliferation via β-catenin and MAPK pathway.

## Discussion

PTOV1 expression predicts the poor prognosis in multiple tumors [9, 10, 38, 39]. Studies on the interacting partners and the proteins that share the close regulatory network with PTOV1 has helped to elucidate the cellular functions. Our study has taken the first step in establishing the functional relationship between PIN1 and PTOV1. On the assumption that functionally related genes show a similar expression profile and could fall in the same pathway, in our study we have used the RNAi mediated approach to knock down the genes and explore the different parameters of cell transformation such as cell proliferation, migration, cell cycle, autophagy and apoptosis that are required to draw the correlation between the genes. Our earlier finding showed that PTOV1 physically interacts with PIN1. PTOV1 also regulates the expression of PIN1 substrate c-Jun phosphorylation which is a major transcription factor involved in cell proliferation, migration, and apoptosis [8].

Cell viability and colony formation of MDA-MB-231 and MCF-7 cells were significantly reduced after PIN1 and PTOV1 knockdown, which suggests that both the genes have a strong impact on the growth of cells. Silencing of PIN1 and PTOV1 strongly inhibited the migration of cells. The role of PTOV1 in cell cycle progression has been studied in prostate cancer cells [38]. There are numerous reports on the role of PIN1 in cell cycle [39, 40]. Here, we have shown that PIN1 and PTOV1 knockdown arrests breast cancer cell MDA-MB-231 in the G2/M phase of the cell cycle before entry into the mitosis where rapid synthesis of proteins is required for cell division. This supports the role of PTOV1 in protein translation.

Modulation of redox status can be employed for the killing of cancer cells [16]. We found that PIN1 and PTOV1 both hold promising target to change the redox status of cancer cells. The balance between cell proliferation and regression of tumor determines the efficacy of radiotherapy, chemotherapy or hormonal therapy, which is governed, in part, by the repertoire of pro-apoptotic and anti-apoptotic molecules [41–45]. Here, we have studied the expression of Bcl-2 and Bcl-xL (anti-apoptotic) and BAX (pro-apoptotic) after knocking down of our target genes. Downregulation of Bcl-2, Bcl-xL and up-regulation of BAX confirmed that cells were driven towards apoptosis after the knocking down of PIN1 and PTOV1 respectively. Therapeutic targeting for autophagy induction is a newer form of cell death in cancer which depends on cell type [46]. Knockdown of PTOV1 increased the expression of autophagy markers Beclin-1 and LC3, suggesting that cell death also induced by autophagy.

Both PIN1 and PTOV1 knockdown regulates multiple genes involved in MDA-MB-231 cell proliferation, cell cycle, metastasis, and apoptosis in their transcriptional level. Western blot analysis was performed to study the expression of different proteins related to cell transformation. First, we studied the interaction between PIN1 and PTOV1in MDA-MB-231 cells by co-immunoprecipitation. We found that PTOV1 was enriched in the PIN1 complex. Silencing of PIN1 downregulated the expression of PTOV1 but not vice-versa. Treatment with Juglone completely blocked the expression of PTOV1. Also, the ectopic expression of PIN1 increased the expression of PTOV1. Taken together, all these results show that PIN1 is upstream of PTOV1 in the signaling cascade. Activation of Wnt/β-catenin is a good predictor with poor prognosis in breast cancer [47, 48]. PIN1 and PTOV1 downregulation inhibited the expression of β-catenin. This result showed that PIN1 and PTOV1 might be linked for the activation of Wnt/β-catenin signaling pathway. Our results show that PIN1 and PTOV1 target Cyclin D1 and c-Myc. Extracellular signal-regulated kinases (ERKs), one of the members of mitogen-activated protein kinases (MAPKs), are the substrates for proline-directed phosphorylation by PIN1[37]. Silencing of PTOV1 also abolished the phosphorylation of ERK. It can be speculated that PTOV1 may be required for the PIN1 mediated phosphorylation of ERK. RACK1 has earlier been shown to interact with PTOV1 which regulates translation of c-Jun in PC-3 cells [10]. Our previous finding showed that PTOV1 is a novel regulator of PIN1 mediated c-Jun phosphorylation and PTOV1 interacts with PIN1 [8]. Here, we have shown that RACK1 is regulated by both PIN1 and PTOV1. Summing up all these results, it can be deduced that PTOV1 is contributing for PIN1 mediated cell transformation.

**Fig 7:**
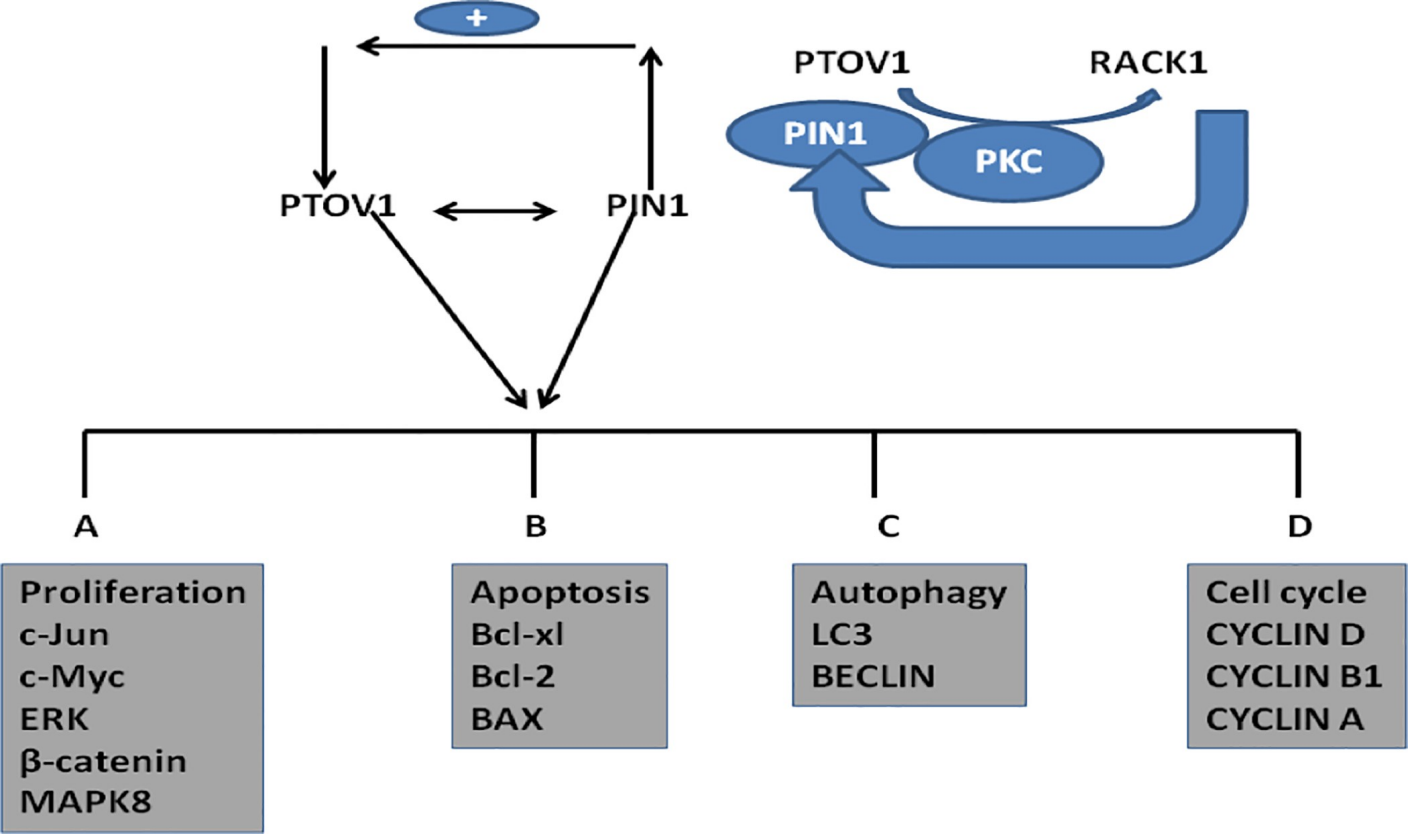
PIN1& PTOV1interaction. Overexpression of PIN1 induces the expression of PTOV1, which suggest that PIN1 might be the upstream protein which regulates the expression of PTOV1. Both the gene cooperatively share MAPK pathway including the expression of ERK, C-Jun and the master regulator C-Myc as well as induces the expression of apoptosis and autophagy related genes. PTOV1 interacts with RACK1 which is involved for protein synthesis in cancer similarly knockdown of PIN1 also inhibits RACK1 and thus PIN1 might contribute to oncogenic signalling via PTOV1-RACK1 interaction.

In conclusion, we have demonstrated that PIN1 and PTOV1 show similar expression profiles of a cohort of genes by RNAi silencing in MDA-MB-231 cells. Functional validation of their relationship was found to be both at transcriptional and translational levels and showed their effect on cell viability, colony forming potential, cell migration, cell cycle, and metastasis.PIN1 and PTOV1 drives the expression of Cyclin D1 c-Myc & β-catenin. Also silencing of genes affect the proteins related to MAPK pathway. Both of genes affect the overall survival of MDA-MB-231 cells by targeting BAX, Bcl-2, and Bcl-xL as shown in Fig 6. Thus, both PIN1 and PTOV1 drive the common genes regulation to impart oncogenic phenotype of MDA-MB-231 cells, thus PIN1- PTOV1 complex could be a potential target for future cancer therapy as shown in Fig 7.

## Supporting information

S1 Fig Silencing of PIN1 and PTOV1 decrease the proliferation, colony formation, and migration of MCF-7 cells. (DOCX)

S1 File (DOCX)
