# Supplementary material for: Knockdown of PTOV1 and PIN1 exhibit common phenotypic anti-cancer effects in MDA-MB-231 cells
Source: PLoS One. 2019 May 13;14(5):e0211658. doi: 10.1371/journal.pone.0211658 (PMC6513092; doi:10.1371/journal.pone.0211658)

Manuscript: PONE-D-19-01352

**Knockdown of PTOV1 and PIN1 Exhibit Common Phenotypic Anti-Cancer Effects in MDA-MB-231 Cells**

Shibendra Kumar Lal Karna, Faiz Ahmad, Bilal Ahmad Lone & Yuba Raj Pokharel^*^

Cancer Biology Laboratory, Faculty of Life Science & Biotechnology, South Asian University, Akbar Bhawan, Chankyapuri, New Delhi 110021, India

^*^Corresponding author: Yuba Raj Pokharel, [yrp@sau.ac.in](mailto:yrp@sau.ac.in)

Data Share from the conclusion drawn for this manuscript


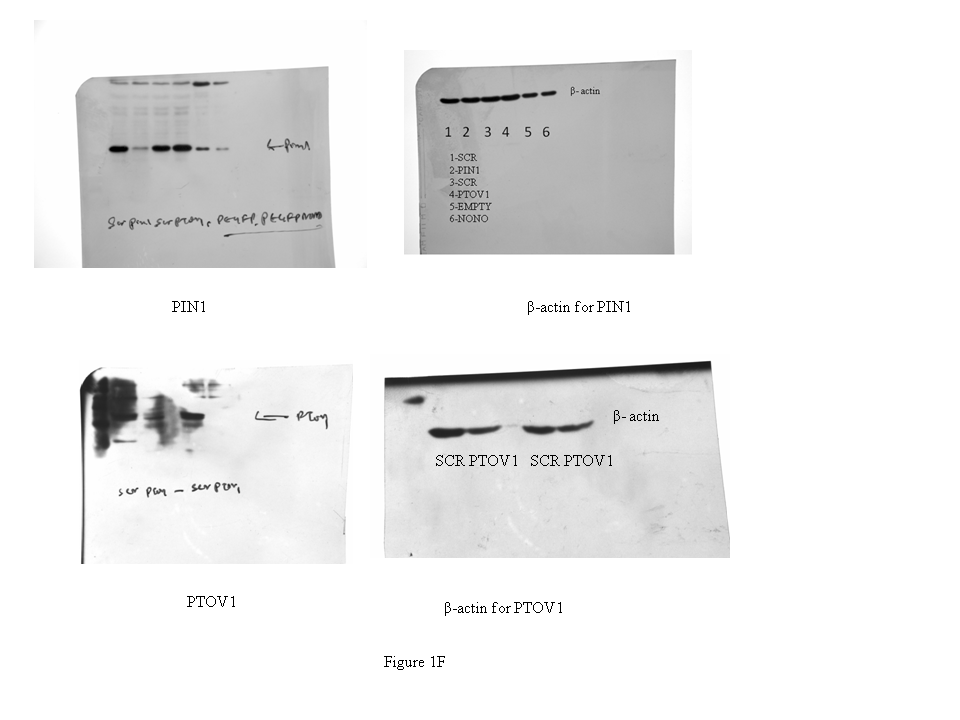


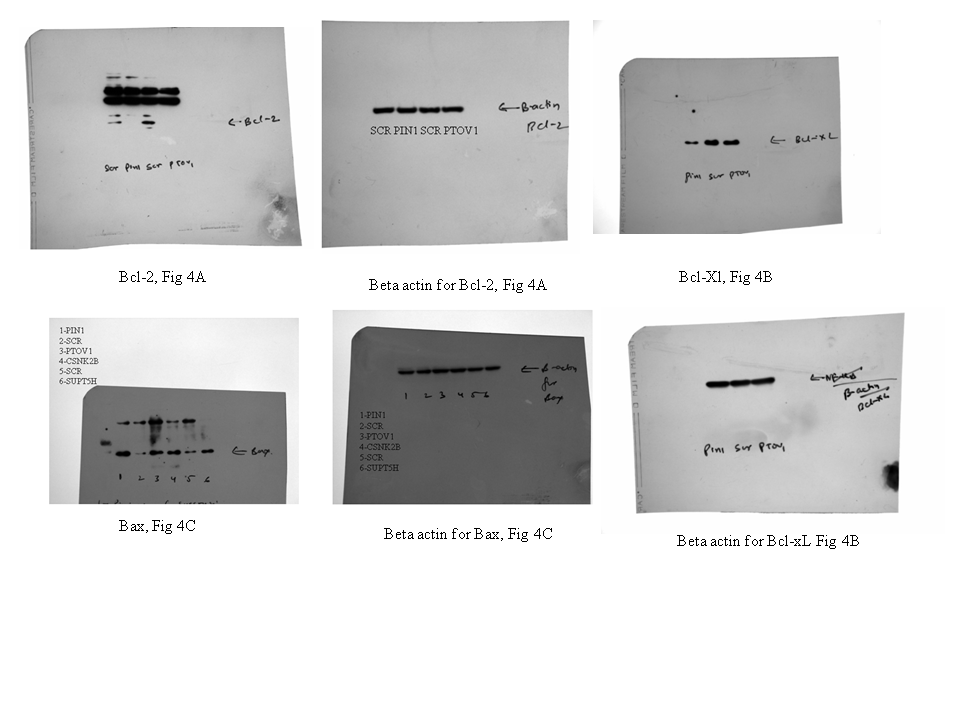


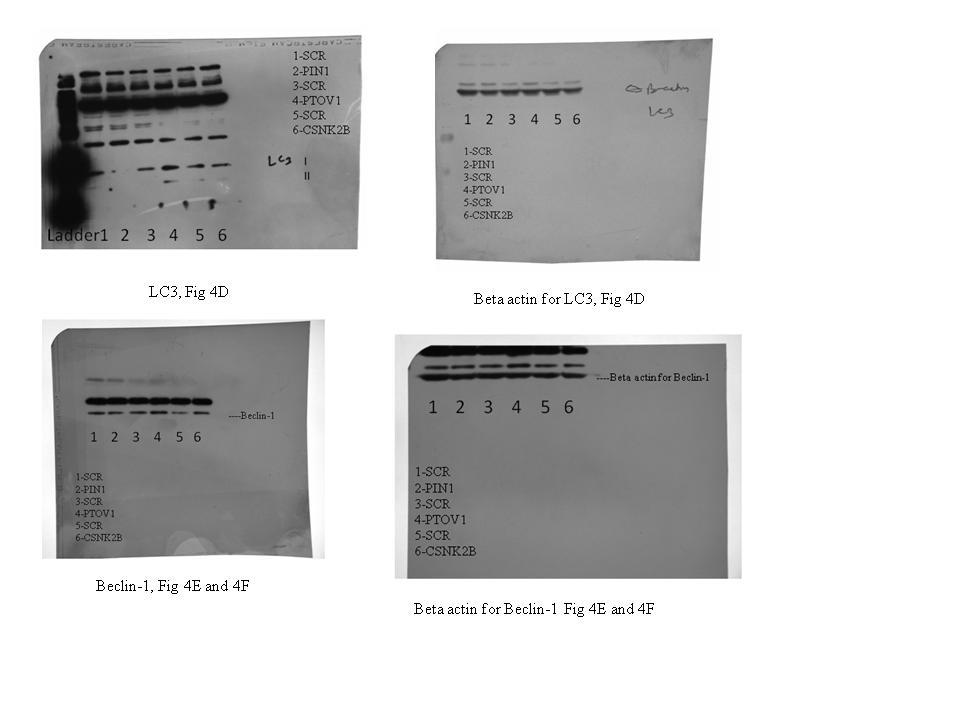


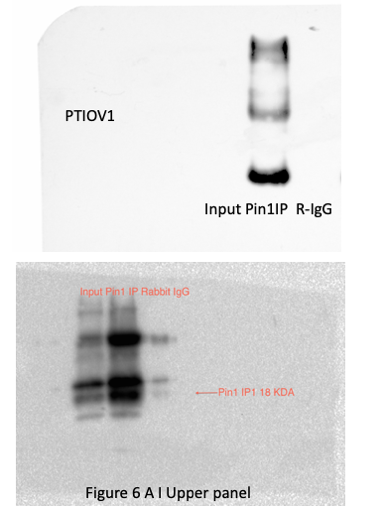


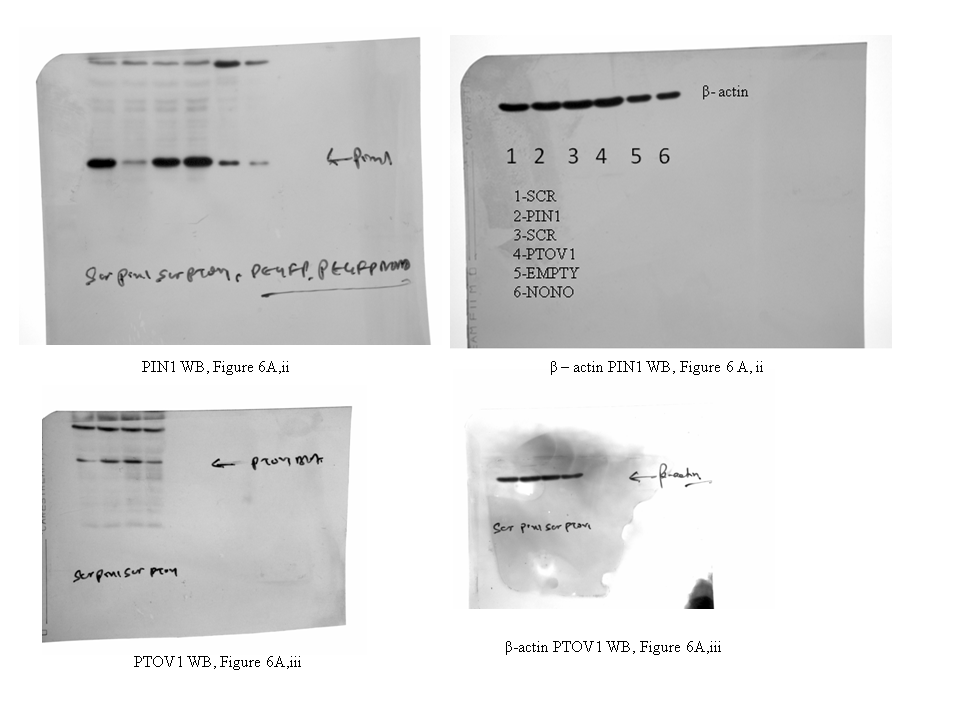


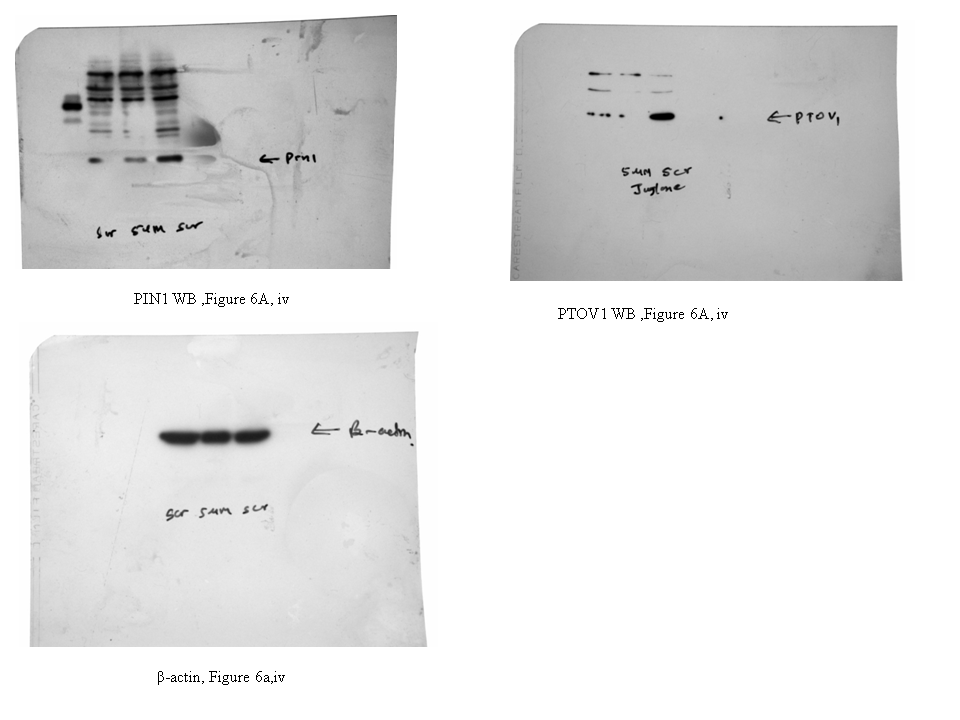


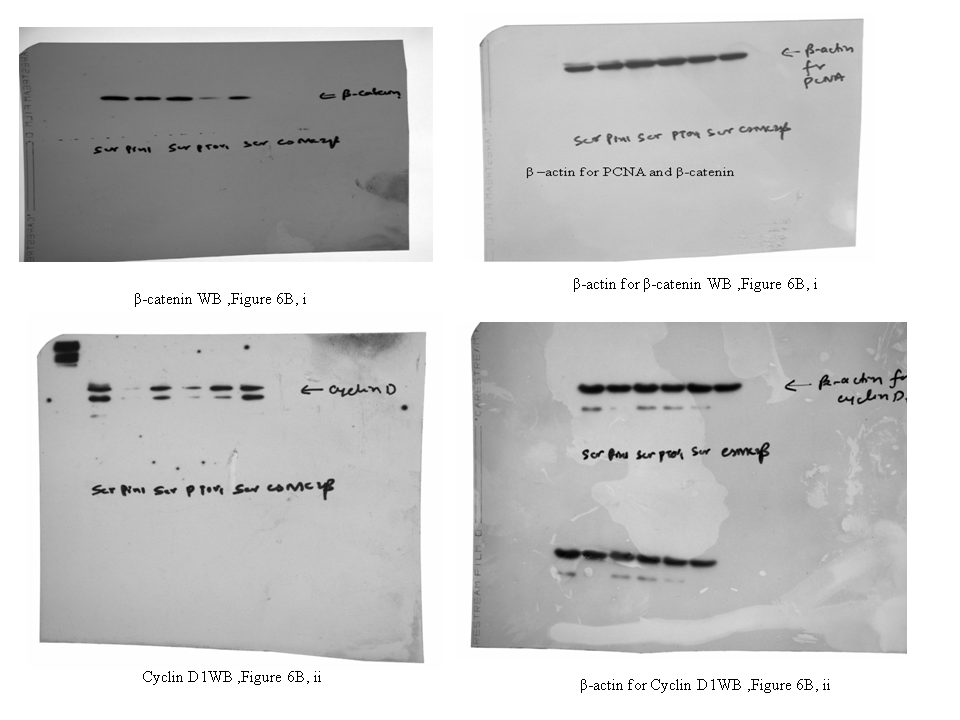


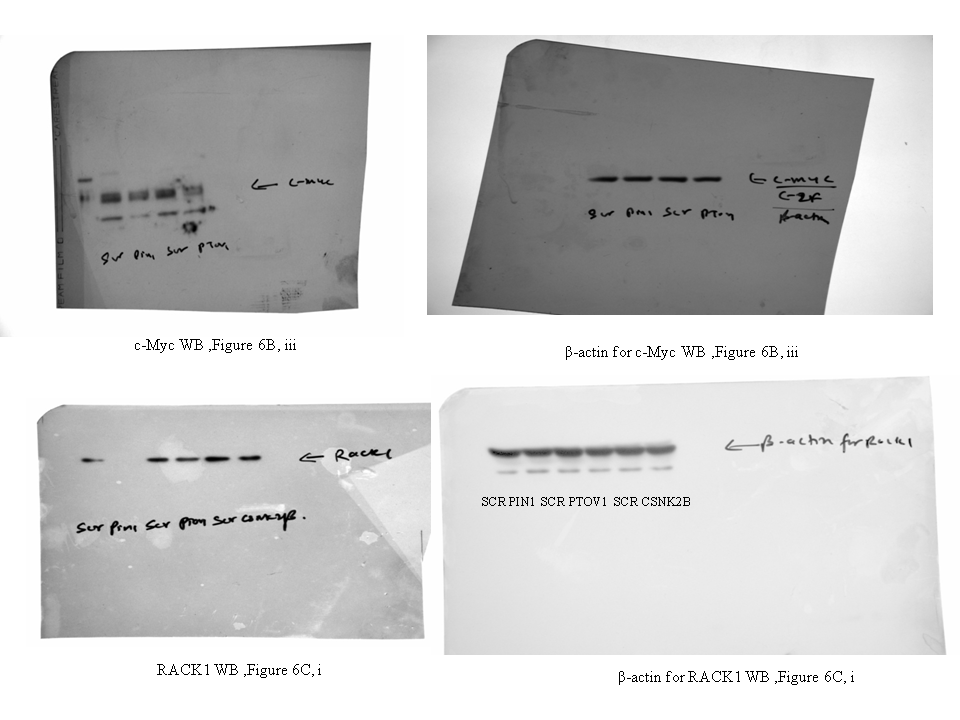


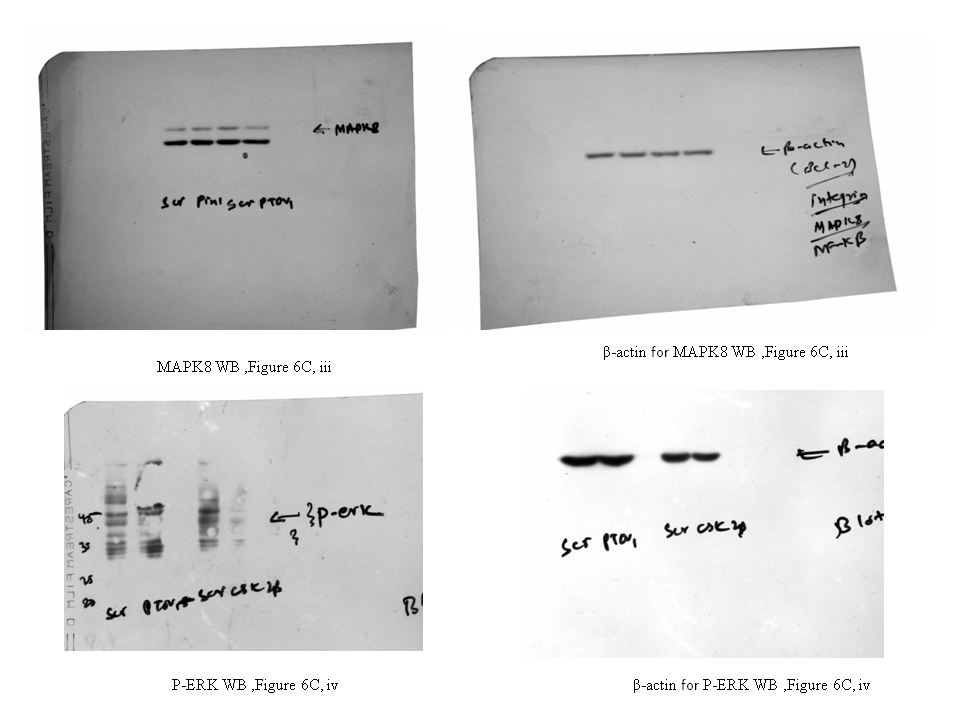

Supplement: S1 Fig — (DOCX) [file pone.0211658.s001.docx]
