# Supplementary material for: Knockdown of PTOV1 and PIN1 exhibit common phenotypic anti-cancer effects in MDA-MB-231 cells
Source: PLoS One. 2019 May 13;14(5):e0211658. doi: 10.1371/journal.pone.0211658 (PMC6513092; doi:10.1371/journal.pone.0211658)
Supplement: S1 File — (DOCX) [file pone.0211658.s002.docx]

**Knockdown of PTOV1 and PIN1 Share Common Genes Regulation in MDA-MB-231 Cells *in vitro***

Shibendra Kumar Lal Karna, Faiz Ahmad, Bilal Ahmad Lone & Yuba Raj Pokharel^*^

Cancer Biology Laboratory, Faculty of Life Science & Biotechnology, South Asian University, Akbar Bhawan, Chankyapuri, New Delhi 110021, India

^*^Corresponding author: Yuba Raj Pokharel, yrp@sau.ac.in


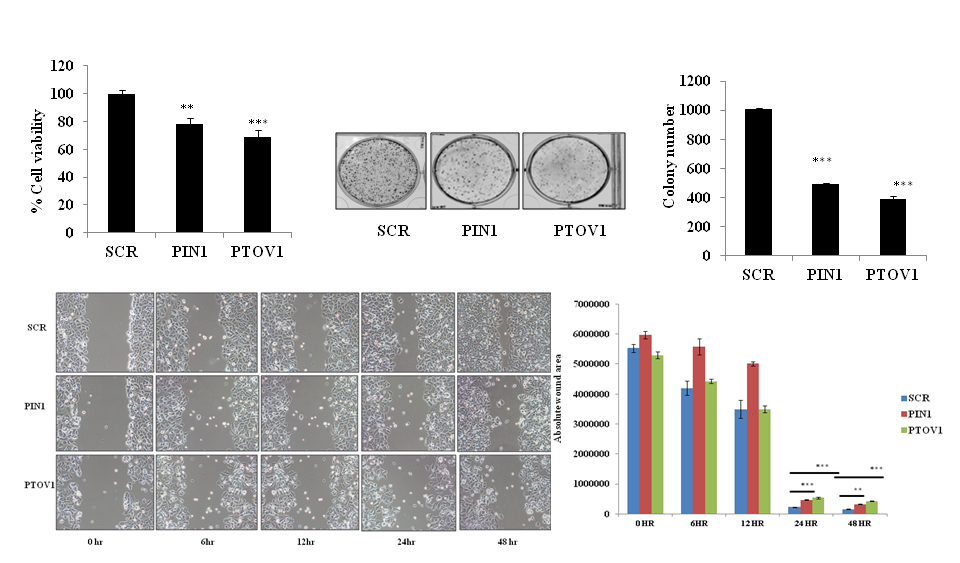


**A**

**B**

**C**

**D**

**E**

**Figure S1. Silencing of PIN1 and PTOV1 decrease the proliferation, colony formation, and migration of MCF-7 cells.** A. Cell viability was estimated by using crystal violet assay and absorbance signal was recorded in microtiter plate ELISA reader at 570 nm. (B) Representative images of the colony of cells transfected with siRNAs in 6 well plates after 2 weeks of incubation. (C) Densitometry representation of colony number using Image J software. (D) Representative images of the wound area of each treated group after 0, 6, 12, 24 and 48 hours of scratch. (E) Densitometry representation of Wound area using Image J software. Data are represented as mean ± SD of three independent experiments. ** and *** Significant difference from scramble groups (p < 0.01 & 0.001 respectively).
